# Letters from Far Japan

**Published:** 1892-02-06

**Authors:** Ernest Hart


### Letters from Far Japan.

By Mrs. Ernest Hart.

### II.—HOSPITALS AND MEDICAL EDUCATION IN JAPAN.—Part I.

It was only just twenty-four years ago, in 1868, that the Japanese resolved to renounce feudalism and to adopt European civilization, methods of defence, and scientific education. In the single generation which has elapsed since this great resolution was taken the advance made has been prodigious. In place of a fleet of unwieldly junks Japan has now a well-equipped navy, with torpedoes, gunboats, and ironclads; instead of the bands of picturesque Samarai, dressed in quaint and artistic armour and stiff draperies of silk damask, there is now a well-drilled army, organized on the German model; and, reversing the policy of rigid exclusion of all the ideas, arts, and sciences of the "hairy barbarians," there is now an elaborate educational system established throughout Japan on modern principles, and the young Japanese students have shown themselves passionately eager to master and make their own the latest achievements of science and civilization. Space prevents us from doing more than notice the medical and science departments of the University of Tokio. The University sprang from an old institution founded by the Tokuyara surgeons for the study of barbarian languages. It was reorganised on European models soon after the Restoration, in 1868. It consists of five faculties, those of law, literature, science, engineering, and medicine. The medical school is thoroughly equipped with professors, assistant professors, lecturers, laboratories, library, &c. The language used is German. In studying and teaching modern science it has been found impossible to adapt the Japanese language to the idioms, phraseology, and etymology of science. German or English has therefore been adopted as the language of tuition and expression in scientific matters, and as most of the Japanese professors obtained their medical education and degrees in the Universities of Leipsic, Heidelberg, Berlin, and Strasbourg, German is the language in which they teach. With two exceptions, the whole of the medical school is now officered by Japanese professors, the Director being Professor Hüdzu Miyake, who speaks German and English equally fluently. The course of study extends over four years, and embraces anatomy, physiology, histology, pathology, materia medica, ophthalmology, clinical medicine and surgery, gynæcology, hygiene, and other subjects. The education given is said to be rather over-scientific and not sufficiently practical; but in the clinical examinations it is noticeable the student is not merely expected to make a diagnosis of a case in the wards, but is required to treat one or two patients for a week, to keep during this time a complete clinical journal, and give at the end a clearly written statement of his opinion of the case to the Committee of Examination.

There are two hospitals in the University grounds, where students study in the wards and carry out investigations on subjects relating to the sciences of medicine and surgery in the laboratories attached. The principal hospital contains 192 beds situated in 120 rooms, and there is also an extensive out-patient department. The second, or Shitaya Hospital, is purely for out-patients, and to it are sent the cases which are considered to be specially instructive to the students. These hospitals are pay hospitals, and there are three scales of charges for patients, according to the difference of room and accommodation, no charge being, however, made to poor patients for medical attendance and necessaries. The beds being occupied by paying patients it results that there are not the same facilities given for clinical study as in free hospital which are supported by the State or charity.

The medical students must live within the compounds of the University, in dormitories specially built and arranged for this purpose, or in authorised boarding-houses, or, by special permission, at home or in the house of a professor. They are subject to the strictest discipline and surveillance. I was, however, assured that stimulation to study was not needed, so eager were the students to learn and make their own all science had to give. There are three kinds of students, under-graduates, elective students, and post-graduate students. The elective students are graduates of any medical school recognised by the State, and who come to the University to follow certain courses or to study in the laboratories. For the post-graduate students special opportunities of study are given. The tuition fee is $2.50 (7s. 9d.) a month, paid monthly during term time. The cost of the living expenses in the dormitories is from $7.50 (23s.) to $12 (37s.) a month. There are seven laboratories for anatomy, physiology, pathology, pharmacology, hygiene, forensic medicine, and pharmacy in the College of Medicine.

A number of medical journals are published in Japan of a very high order of merit. They are written in Japanese, German, and English, and are often beautifully illustrated.

*(To be continued.)*

